# Associations with intraocular pressure across Europe: The European Eye Epidemiology (E^3^) Consortium

**DOI:** 10.1007/s10654-016-0191-1

**Published:** 2016-09-09

**Authors:** Anthony P. Khawaja, Henriët Springelkamp, Catherine Creuzot-Garcher, Cécile Delcourt, Albert Hofman, René Höhn, Adriana I. Iglesias, Roger C. W. Wolfs, Jean-François Korobelnik, Rufino Silva, Fotis Topouzis, Katie M. Williams, Alain M. Bron, Gabriëlle H. S. Buitendijk, Maria da Luz Cachulo, Audrey Cougnard-Grégoire, Jean-François Dartigues, Christopher J. Hammond, Norbert Pfeiffer, Angeliki Salonikiou, Cornelia M. van Duijn, Johannes R. Vingerling, Robert N. Luben, Alireza Mirshahi, Julia Lamparter, Caroline C. W. Klaver, Nomdo M. Jansonius, Paul J. Foster, On behalf of the European Eye Epidemiology (E³) Consortium, Niyazi Acar, Eleftherios Anastosopoulos, Augusto Azuara-Blanco, Arthur Bergen, Geir Bertelsen, Christine Binquet, Alan Bird, Lionel Brétillon, Alain Bron, Gabrielle Buitendijk, Maria Luz Cachulo, Usha Chakravarthy, Michelle Chan, Petrus Chang, Annemarie Colijn, Audrey Cougnard-Grégoire, Catherine Creuzot-Garcher, Philippa Cumberland, José Cunha-Vaz, Vincent Daien, Gabor Deak, Cécile Delcourt, Marie-Noëlle Delyfer, Anneke den Hollander, Martha Dietzel, Maja Gran Erke, Sascha Fauser, Robert Finger, Astrid Fletcher, Paul Foster, Panayiota Founti, Arno Göbel, Theo Gorgels, Jakob Grauslund, Franz Grus, Christopher Hammond, Catherine Helmer, Hans-Werner Hense, Manuel Hermann, René Hoehn, Ruth Hogg, Frank Holz, Carel Hoyng, Nomdo Jansonius, Sarah Janssen, Anthony Khawaja, Caroline Klaver, Jean-François Korobelnik, Julia Lamparter, Mélanie Le Goff, Sergio Leal, Yara Lechanteur, Terho Lehtimäki, Andrew Lotery, Irene Leung, Matthias Mauschitz, Bénédicte Merle, Verena Meyer zu Westrup, Edoardo Midena, Stefania Miotto, Alireza Mirshahi, Sadek Mohan-Saïd, Alyson Muldrew, Michael Mueller, Sandrina Nunes, Konrad Oexle, Tunde Peto, Stefano Piermarocchi, Elena Prokofyeva, Jugnoo Rahi, Olli Raitakari, Luisa Ribeiro, Marie-Bénédicte Rougier, José Sahel, Aggeliki Salonikiou, Clarisa Sanchez, Steffen Schmitz-Valckenberg, Cédric Schweitzer, Tatiana Segato, Jasmin Shehata, Rufino Silva, Giuliana Silvestri, Christian Simader, Eric Souied, Henriet Springelkamp, Robyn Tapp, Fotis Topouzis, Virginie Verhoeven, Therese Von Hanno, Stela Vujosevic, Katie Williams, Christian Wolfram, Jennifer Yip, Jennyfer Zerbib, Isabella Zwiener

**Affiliations:** 1 Department of Public Health and Primary Care, Institute of Public Health, University of Cambridge School of Clinical Medicine, Cambridge, UK; 2 Department of Ophthalmology, Erasmus Medical Center, Rotterdam, The Netherlands; 3 Department of Epidemiology, Erasmus Medical Center, Rotterdam, The Netherlands; 4 Department of Ophthalmology, University Hospital, 21000 Dijon, France; 5 Univ. Bordeaux, ISPED, 33000 Bordeaux, France; 6 Centre INSERM U897-Epidemiologie-Biostatistique, INSERM, 33000 Bordeaux, France; 7 Netherlands Consortium for Healthy Ageing, Netherlands Genomics Initiative, The Hague, The Netherlands; 8 Department of Ophthalmology, University Medical Center Mainz, Mainz, Germany; 9 Department of Ophthalmology, Inselspital, Bern, Switzerland; 10 Service d’Ophtalmologie, CHU de Bordeaux, Bordeaux, 33000 France; 11 Faculty of Medicine, University of Coimbra (FMUC), Coimbra, Portugal; 12 Department of Ophthalmology, Centro Hospitalar e Universitário de Coimbra (CHUC), Coimbra, Portugal; 13 Association for Innovation and Biomedical Research on Light and Image (AIBILI), Coimbra, Portugal; 14 A Department of Ophthalmology, Aristotle Universty of Thessaloniki, Thessaloniki, Greece; 15 Departments of Ophthalmology and Twin Research, King’s College London, London, UK; 16 Dardenne Eye Hospital, Bonn, Germany; 17 Department of Ophthalmology, University Medical Center Groningen, University of Groningen, Groningen, The Netherlands; 18 NIHR Biomedical Research Centre, Moorfields Eye Hospital NHS Foundation Trust and UCL Institute of Ophthalmology, London, UK; 19 Moorfields Eye Hospital NHS Foundation Trust, 162 City Road, London, EC1V 2PD UK

**Keywords:** Intraocular pressure, Epidemiology, Body mass index, Refractive errors, Blood pressure, Glaucoma

## Abstract

Raised intraocular pressure (IOP) is the most important risk factor for developing glaucoma, the second commonest cause of blindness globally. Understanding associations with IOP and variations in IOP between countries may teach us about mechanisms underlying glaucoma. We examined cross-sectional associations with IOP in 43,500 European adults from 12 cohort studies belonging to the European Eye Epidemiology (E^3^) consortium. Each study conducted multivariable linear regression with IOP as the outcome variable and results were pooled using random effects meta-analysis. The association of standardized study IOP with latitude was tested using meta-regression. Higher IOP was observed in men (0.18 mmHg; 95 % CI 0.06, 0.31; *P* = 0.004) and with higher body mass index (0.21 mmHg per 5 kg/m^2^; 95 % CI 0.14, 0.28; *P* < 0.001), shorter height (−0.17 mmHg per 10 cm; 95 % CI –0.25, −0.08; *P* < 0.001), higher systolic blood pressure (0.17 mmHg per 10 mmHg; 95 % CI 0.12, 0.22; *P* < 0.001) and more myopic refraction (0.06 mmHg per Dioptre; 95 % CI 0.03, 0.09; *P* < 0.001). An inverted U-shaped trend was observed between age and IOP, with IOP increasing up to the age of 60 and decreasing in participants older than 70 years. We found no significant association between standardized IOP and study location latitude (*P* = 0.76). Novel findings of our study include the association of lower IOP in taller people and an inverted-U shaped association of IOP with age. We found no evidence of significant variation in IOP across Europe. Despite the limited range of latitude amongst included studies, this finding is in favour of collaborative pooling of data from studies examining environmental and genetic determinants of IOP in Europeans.

**Electronic supplementary material:**

The online version of this article (doi:10.1007/s10654-016-0191-1) contains supplementary material, which is available to authorized users.

## Introduction

Glaucoma is the second commonest cause of blindness globally following cataract, accounting for 8 % of world blindness [1]. Raised intraocular pressure (IOP) is an important risk factor for the incidence [2] and progression [3] of the commonest form of glaucoma, primary open-angle glaucoma (POAG). Understanding which systemic and ocular parameters are associated with IOP gives us insight into the pathophysiological mechanisms underlying IOP and may ultimately lead to new targets or treatment methods for POAG. Examining geographic trends in disease may also shed light on disease risk and aetiology. For example, differential rates of coronary heart disease mortality across Europe gave impetus to research demonstrating a beneficial effect of a Mediterranean diet [4].

Several European population studies have reported IOP data [5–9]. However, individual studies suffer from limited sample size and results may only apply to the geographical region examined. We therefore conducted a study of IOP data from 12 population-based studies across Europe, maximising power to detect small associations and increasing generalisability to European populations. We also aimed to compare IOP between studies, in particular comparing IOP in studies from Southern Europe with IOP in studies from more northern Europe (including Northern, Central and Western Europe), potentially reflecting differences in lifestyle, such as diet [10], as well as latitude.

## Methods

The European Eye Epidemiology (E^3^) consortium is a collaborative network of 38 population-based studies across Europe with the overarching aim of developing and analysing large pooled datasets to increase understanding of eye disease and vision loss [11]. Data on IOP were available from 12 E^3^ studies from 6 countries (Table 1). All data from contributing studies were cross-sectional in nature and if multiple IOP measurements were taken per participant, these were measured on the same day. Detailed methods for the studies are given in Supplementary Section A. All studies adhered to the tenets of the Declaration of Helsinki and had local ethical committee approval. All participants gave written informed consent.

**Table 1:** Descriptive data for contributing studies

Study	Years	City/country	IOP measurement		N	Women (%)	Mean age in years (SD)	Mean IOP in mmHg (SD)
			Type	Details				
Alienor Study [37]	2006–2008	Bordeaux, France	NCT (KT 800, Kowa)	1 measurement by a trained technician	797	55.9	79.1 (4.0)	14.1 (2.4)
Coimbra Eye Study	2009–2011	Coimbra, Portugal	NCT (Nidek Tonoref II)	Mean of ≥3 measurements per eye (up to 5 readings taken if any outliers)	2839	56.6	68.3 (8.2)	14.9 (2.9)
EPIC-Norfolk Eye Study [38]	2004–2011	Norfolk, UK	NCT (ORA)	Best signal value of ≥3 IOPg measurements per eye.	7253	55.1	67.5 (7.4)	16.0 (3.5)
Erasmus Rucphen Family Study [39, 40]	2002–2005	Rucphen, Netherlands	GAT	Median of 3 measurements per eye	2122	56.1	48.2 (14.0)	15.0 (2.9)
Gutenberg Health Study [5]	2007–2012	Mainz, Germany	NCT (Nidek NT-2000)	Mean of 3 measurements per eye	13,600	49.4	54.4 (10.9)	14.2 (2.7)
Montrachet 3C Study	2009–2013	Montrachet, France	NCT (Nidek Tonoref II)	1 measurement by a trained technician	937	58.1	81.3 (3.3)	15.2 (3.1)
POLA Study [41]	1995–1998	Sète, France	GAT	1 measurement by an ophthalmologist	2208	56.1	70.2 (6.5)	14.7 (2.6)
Rotterdam Study I [42]	1993–1995	Rotterdam, Netherlands	GAT	Median of 3 measurements per eye	5198	58.5	69.5 (8.0)	14.5 (2.9)
Rotterdam Study II [42]	2000–2001	Rotterdam, Netherlands	GAT	Median of 3 measurements per eye	2496	54.3	63.9 (7.2)	14.1 (2.9)
Rotterdam Study III [42]	2006–2008	Rotterdam, Netherlands	GAT	Median of 3 measurements per eye	3386	56.5	56.6 (6.3)	13.6 (2.7)
Thessaloniki Eye Study [43]	1999–2005	Thessaloniki, Greece	GAT	Mean of 3 measurements per eye	1993	45.1	70.0 (5.3)	15.0 (3.0)
Twins UK	2001–2014	UK (multiple cities)	NCT (ORA)	Mean of 2 measurements per eye	3252	97.5	56.2 (12.1)	15.5 (3.2)

*EPIC* European Prospective Investigation of Cancer, *GAT* Goldmann applanation tonometry, *IOP* intraocular pressure, *NCT* non-contact tonometry, *ORA* ocular response analyzer, *SD* standard deviation

IOP was measured using Goldmann applanation tonometry (GAT) in 6 studies and non-contact tonometry (NCT) in 6 studies (Table 1). We defined participant IOP as the mean of right and left eye values. Participants with an inter-eye difference in IOP of >6 mmHg were excluded as this may indicate undiagnosed ocular disease or artefact (the 6 mmHg cut-off was based on approximately twice the standard deviation).

Factors to be tested for association with IOP were decided a priori, based on common measures available in all studies with IOP data available; these were age, sex, height, body mass index (BMI), systolic blood pressure (SBP), refractive error (mean spherical equivalent [SE] of right and left eyes), and history of cataract surgery.

For initial analyses, we excluded participants with a history of a glaucoma therapy (laser, surgery or medication) or intraocular surgery (other than cataract surgery) in either eye. After examining the association of cataract surgery with IOP, we further excluded all participants with a history of cataract surgery, given the strong effect on IOP. Our main analyses were conducted on phakic patients only.

To examine the associations between IOP and the variables of interest, we used linear regression. Primary multivariable models included all the main variables of interest (age, sex, height, BMI, SBP and SE; referred to as “Model 1”). We also further adjusted for central corneal thickness (CCT) in the subset of participants with CCT data available (“Model 2”). There was no evidence for multicollinearity among variables included in the multivariable regression models. For all regression analyses, residuals were plotted and displayed normality. Regression analyses were conducted for each individual study, and then random-effects meta-analysis was used to combine the effect estimates. A random effects approach was decided a priori given the between study heterogeneity in IOP measurement methods. We conducted an influential analysis that examined the contribution of each study to the heterogeneity by sequentially omitting one study and reanalysing the pooled estimate for the remaining studies. We further examined the association between age and IOP, stratified into age groups based on initial results. Additionally, to address the potential bias of participants with the highest IOP being excluded due to using IOP-lowering therapy, we repeated analyses including participants on IOP-lowering medication; for these participants we imputed pre-treatment IOP by dividing measured IOP by 0.7 (“Model 3”). This approach assumes an average IOP reduction of 30 % on medical treatment and has been used successfully in the study of genetic associations with IOP [12]. For the Coimbra Eye Study, data on SBP were not available and multivariable adjusted effect estimates were adjusted for age, sex, height, BMI and SE only; we therefore conducted sensitivity analyses of excluding the Coimbra Eye Study from the meta-analyses. Regression analyses for data from the Twins UK study included data from both twins in each pair and therefore used a clustered analysis approach to account for any correlation between twins. We explored the shapes of the associations with IOP by plotting random effects meta-analyzed IOP levels with 95 % confidence intervals by ordinal categories of the variables.

Comparing raw mean IOP values between studies is problematic given the different distribution of IOP-associated parameters across the studies. We therefore calculated a standardized IOP for each study using multivariable linear regression, based on fixed covariable parameters; these parameters were set to values likely to be included within the range values of values for each study (age 65 years, sex 1.5, SBP 135 mmHg, height 165 cm, BMI 25 kg/m^2^, SE 0). To compare IOP in different regions in Europe, we divided the studies into “northern” and “southern” groups using an arbitrary latitude cut-point of 50° to derive two similarly sized groups (i.e. the definitions of “northern” and “southern” are based on dividing the included studies into two groups rather than being representative of geographic regions in Europe). We used random-effects meta-analysis to derive pooled standardized IOP estimates, and these were compared using the independent samples *t* test. We examined the association between standardized IOP and latitude as a continuous variable using meta-regression. We also compared standardized IOP in GAT studies with standardized IOP in NCT studies, and further examined the association between latitude and standardized IOP stratified by tonometry method.

Stata version 13.1 (StataCorp LP, College Station, TX) was used for all analyses.

## Results

A total of 46,081 participants from 12 population-based studies were included. The mean age of participants ranged from 49 to 81 years, and 57 % were women (Table 1). Mean IOP ranged from 13.6 mmHg in the Rotterdam Study III to 16.0 mmHg in the EPIC-Norfolk Eye Study (Table 1).

**Fig. 1:**
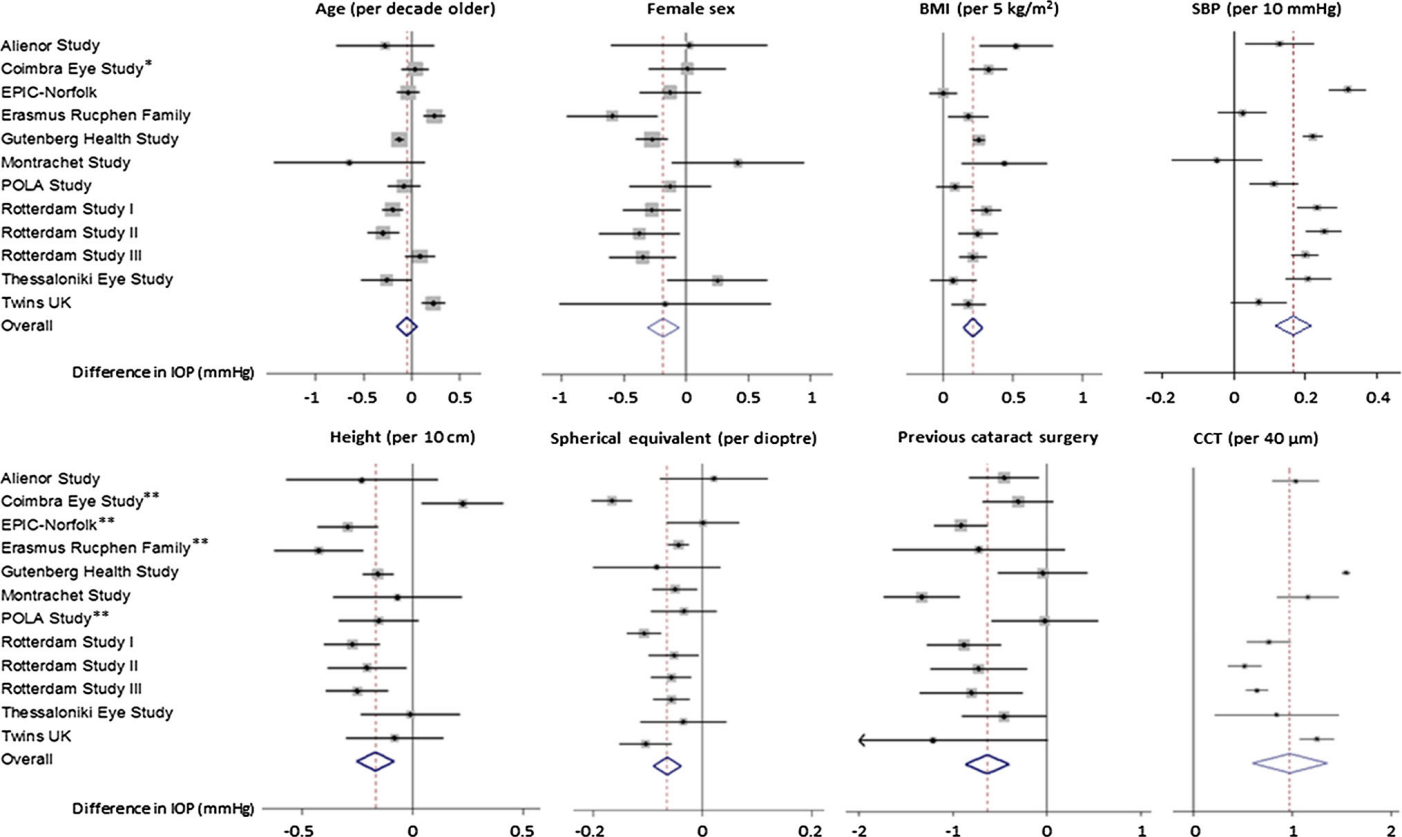
Forest plots for associations with intraocular pressure (IOP). All associations were adjusted for age, sex, body mass index (BMI), systolic blood pressure (SBP), height and spherical equivalent unless otherwise indicated. Results are for phakic participants (n = 43,500) except for cataract surgery (phakic and pseudophakic, n = 46,081) and CCT (n = 21,332 with complete data). *Single asterisk* SBP was not measured or adjusted for in the Coimbra Eye Study. *Double asterisks* CCT was not measured in these studies

In total, 2581 participants (5.6 %) had undergone cataract surgery in at least one eye; on average, these participants had 0.61 mmHg lower IOP (Table 2). All but four studies had CCT measurements available (Fig. 1). On average, IOP was measured 0.96 mmHg higher per 40 µm thicker CCT (Table 2). For subsequent analyses, we excluded participants with a history of cataract surgery; results below refer to a total of 43,500 phakic participants for primary analyses and 21,332 participants with CCT data also available for further adjustment.

**Table 2:** Meta-analyzed associations with intraocular pressure (IOP)

	Unadjusted		Model 1		Model 2	
	Difference in IOP (95 % CI), mmHg	*P* value	Difference in IOP (95 % CI), mmHg	*P* value	Difference in IOP (95 % CI), mmHg	*P* value
Phakic participants						
Age (per decade)	0.06 (−0.03, 0.16)	0.21	−0.05 (−0.16, 0.06)	0.34	0.00 (−0.19, 0.19)	0.97
Female sex	0.00 (−0.13, 0.13)	1.00	−0.18 (−0.31, −0.06)	**0.004**	−0.04 (−0.20, 0.12)	0.65
BMI (per 5 kg/m^2^)	0.29 (0.22, 0.35)	**<0.001**	0.21 (0.14, 0.28)	**<0.001**	0.25 (0.18, 0.31)	**<0.001**
Height (per 10 cm)	−0.12 (−0.19, −0.04)	**0.003**	−0.17 (−0.25, −0.08)	**<0.001**	−0.14 (−0.25, −0.04)	**0.008**
SBP (per 10 mmHg)	0.19 (0.15, 0.23)	**<0.001**	0.17 (0.12, 0.22)	**<0.001**	0.19 (0.13, 0.25)	**<0.001**
Spherical equivalent (per dioptre)	−0.04 (−0.07, −0.01)	**0.007**	−0.06 (−0.09, −0.03)	**<0.001**	−0.07 (−0.08, −0.06)	**<0.001**
Phakic and pseudophakic participants						
Cataract surgery^a^	−0.61 (−0.81, −0.41)	**<0.001**	−0.63 (−0.87, −0.40)	**<0.001**	−0.68 (−1.13, −0.23)	**0.003**
Phakic participants with CCT data						
CCT (per 40 µm)^b^	0.96 (0.57, 1.35)	**<0.001**	–	–	0.97 (0.59, 1.35)	**<0.001**

Results are for all phakic participants (n = 43,500), except for cataract surgery (includes pseudophakic participants in addition)^a^ and CCT (a subset of phakic participants)^b^

Unadjusted—results are from univariable regression models

Model 1—results from multivariable regression models adjusted for age, sex, body mass index (BMI), height, systolic blood pressure (SBP) and spherical equivalent

Model 2—adjusted for central corneal thickness (CCT) in addition to covariables adjusted for in Model 1 (n = 21,332)

^a^Analyses carried out on data from phakic and pseudophakic participants (n = 46,081 for unadjusted and Model 1; n = 21,332 for Model 2)

^b^Analyses carried out on data from 21,332 phakic participants with complete data for CCT in addition to other covariables

*P* values < 0.05 are in bold

Table 2 presents crude and adjusted meta-analyzed associations with IOP. Figure 1 presents the Forest plots for the meta-analyses adjusted for age, sex, BMI, height, SBP and SE. Age was not significantly associated with IOP in these linear analyses. Sex was only associated with IOP in adjusted analyses; women had 0.18 mmHg lower IOP (*P* = 0.004). Both BMI and SBP were positively associated with IOP in crude and adjusted analyses (all *P* < 0.001). Height was negatively associated with IOP in crude and adjusted analyses (Model 1 *P* < 0.001; Model 2 *P* = 0.008). A more myopic refraction was associated with higher IOP (*P* < 0.001 for adjusted analyses). The *R* ^2^ for IOP in the maximally adjusted multivariable models for each study ranged from 0.09 in the Rotterdam Study II to 0.27 in the Gutenberg Health Study. An influential analysis did not identify one study that consistently contributed to heterogeneity and omitting one study at a time did not meaningfully change any of the results (Supplementary Section B).

**Fig. 2:**
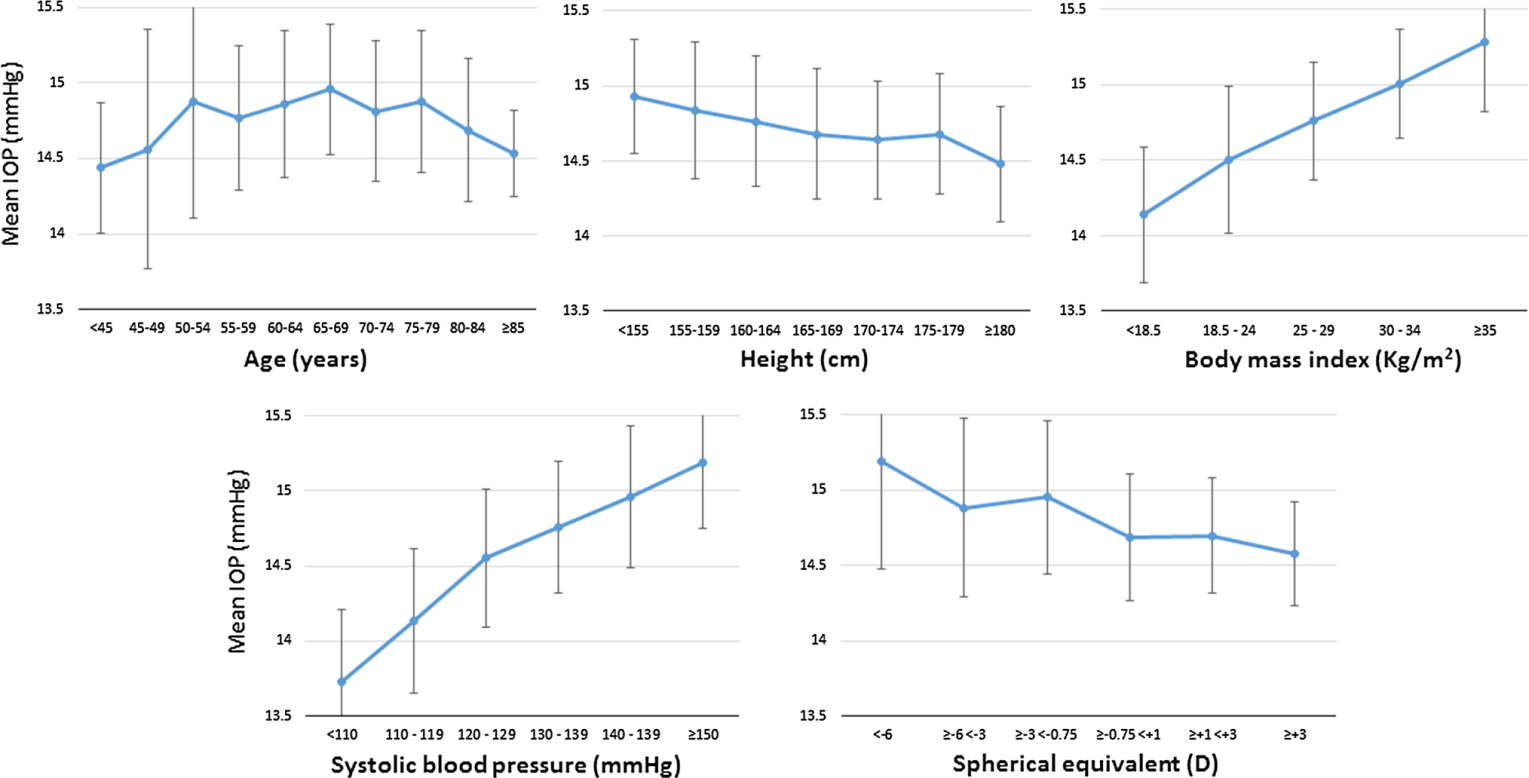
Mean intraocular pressure (IOP) and 95 % confidence intervals plotted for ordinal categories of explanatory variables

Figure 2 illustrates the shapes of the associations with IOP. There were clear linear associations with IOP across the whole ranges of height, BMI, SBP and SE. There was a suggestion of an inverted-U shaped association between age and IOP. To further explore this potential non-linear relationship, we examined the association between age and IOP stratified into 3 age categories (Table 3). We found evidence for increasing IOP with older age in participants under 60 years, though this was only statistically significant for the crude analysis (*P* = 0.005). There was consistent evidence for decreasing IOP with older age in participants 70 years or older (all *P* < 0.01). There did not appear to be a significant relationship between IOP and age for participants aged 60–69 years in primary analyses. To further explore whether the reduction of IOP with increasing age in the oldest participants was due to exclusion of participants with higher IOP following commencement of IOP-lowering medication, we repeated the analysis including participants on IOP-lowering medication and imputing their pre-treatment IOP, and observed similar associations (Table 3, Model 3).

**Table 3:** Associations between age and intraocular pressure (IOP), stratified by age-group

Age group (years)	Unadjusted		Model 1		Model 2		Model 3	
	Difference in IOP per decade older (95 % CI), mmHg	*P* value	Difference in IOP per decade older (95 % CI), mmHg	*P* value	Difference in IOP per decade older (95 % CI), mmHg	*P* value	Difference in IOP per decade older (95 % CI), mmHg	*P* value
<60	0.27 (0.08, 0.46)	**0.005**	0.13 (−0.07, 0.33)	0.22	0.00 (−0.07, 0.07)	0.91	0.28 (0.17, 0.39)	**<0.001**
60–69	0.12 (−0.05, 0.29)	0.16	0.01 (−0.17, 0.19)	0.91	0.24 (−0.27, 0.75)	0.35	0.23 (0.01, 0.45)	**0.038**
≥70	−0.21 (−0.35, −0.07)	**0.003**	−0.28 (−0.44, −0.12)	**0.001**	−0.59 (−1.04, −0.14)	**0.010**	−0.25 (−0.41, −0.09)	**0.002**

Model 1—results from multivariable regression models adjusted for sex, body mass index (BMI), height, systolic blood pressure (SBP) and spherical equivalent (n = 43,500)

Model 2—adjusted for central corneal thickness in addition to covariables adjusted for in Model 1 (n = 21,332)

Model 3—including participants taking IOP-lowering medication (with imputed pre-treatment IOP), adjusted for sex, BMI, height, SBP and spherical equivalent (n = 44,143)

*P* values < 0.05 are in bold

**Fig. 3:**
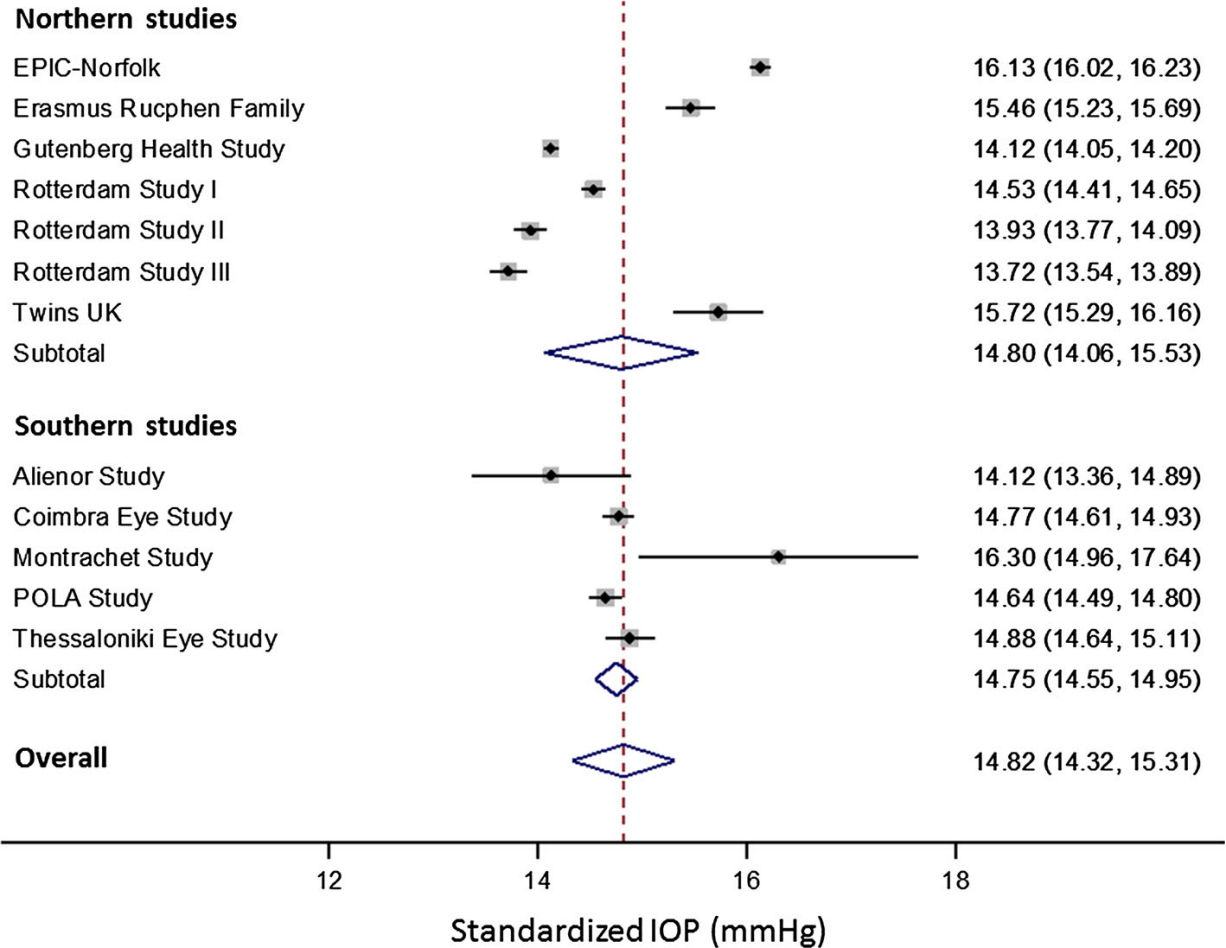
Forest plot of standardized intraocular pressure (IOP), stratified by latitude. Pooled associations for northern studies, southern studies, and overall were derived using random effects meta-analysis. The *right column* presents standardized IOP in mmHg (95 % confidence interval)

**Fig. 4:**
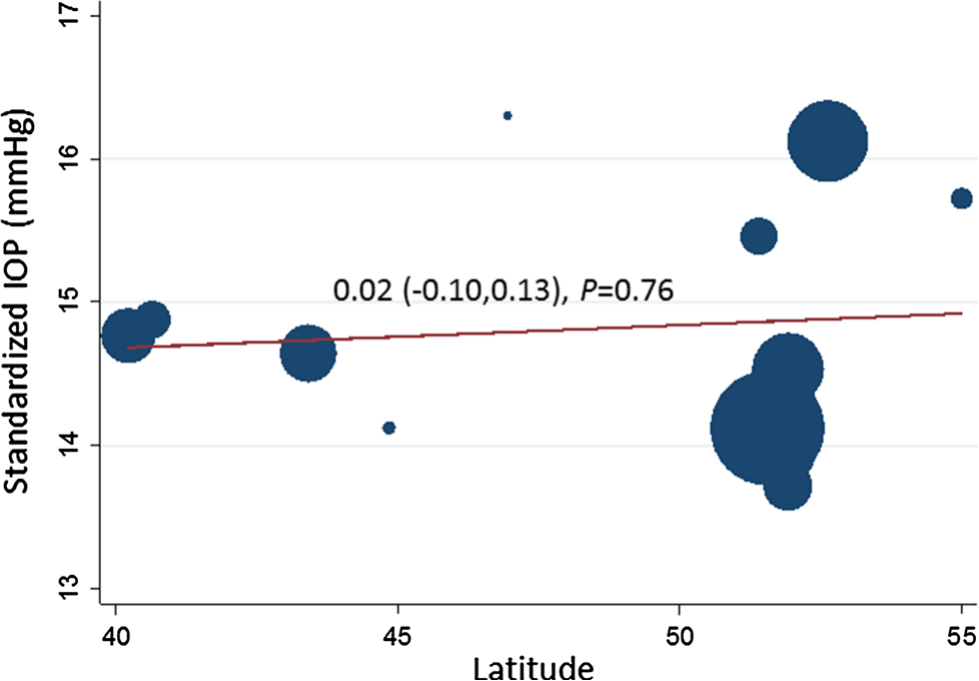
Meta-regression for the association between latitude and standardized intraocular pressure (IOP)

Figure 3 presents the standardized IOP for each country in a Forest plot, stratified by latitude. Standardized IOP varied between 13.7 mmHg in Rotterdam Study III to 16.3 mmHg in the Montrachet Study. The meta-analyzed standardized IOP for all European studies was 14.8 mmHg (95 % CI 14.3, 15.3), and there was no significant difference between northern studies (meta-analyzed IOP 14.80 mmHg) and southern studies (meta-analyzed IOP 14.75 mmHg), as shown in Fig. 3 (*P* = 0.95). We also carried out a meta-regression to examine whether standardized IOP was associated with latitude considered as a continuous variable (Fig. 4); we found no significant association (*P* = 0.76). As shown in Supplementary Figure 1, the standardized IOP for all studies that used NCT (15.2 mmHg; 95 % CI 14.2, 16.2) was higher than the standardized IOP for all studies that used GAT (14.5 mmHg; 95 % CI 14.1, 15.0), though the difference was not statistically significant (*P* = 0.32). We therefore also compared northern versus southern standardized IOP stratified by tonometry method (Supplementary Figure 1); there were no significant differences for either the GAT studies (*P* = 0.56) or the NCT studies (*P* = 0.83). Further, we also carried out meta-regressions using latitude as a continuous variable, stratified by tonometry method (Supplementary Figure 2); there was no significant association for the GAT studies (*P* = 0.51) or the NCT studies (*P* = 0.85).

## Discussion

In this large study examining IOP in over 40,000 participants from six European countries, we confirmed previously reported relationships of IOP with SBP, BMI, refractive error and previous cataract surgery. More novel findings include a negative association between IOP and height and an inverted-U-shaped association between IOP and age. The mean standardized IOP was 14.8 mmHg across all studies, and we did not find any significant geographical trends.

While the IOP-lowering effect of cataract extraction in individuals has been consistently reported in longitudinal surgical case series [13], it is less clear whether people who have undergone cataract surgery have lower IOP than people who have not within a population. The 0.6 mmHg lower IOP we found in pseudophakic compared to phakic participants is significant at a population level, and would translate into around a 10 % reduction in the 5-year incidence of glaucoma based on data from the Rotterdam Study [2], all other factors being equal.

There is no consensus on the direction of association between IOP and age in the literature, with studies reporting increasing IOP [14–17], decreasing IOP [5, 8, 18–21] or no association of IOP [22] with older age. Possible reasons for this inconsistency are differential associations by population, or a non-monotonic relationship between age and IOP such that different studies of different aged participants yield different results. An inverted-U shaped relationship between age and IOP was suggested by data from the Beijing Eye Study, though these results were unadjusted and only certain between group comparisons were statistically significant [23]. We found strong evidence for an inverted-U shaped relationship, with IOP increasing linearly with age up to the age of 60 years, IOP linearly decreasing with age above 70 years, and a plateau with no significant association between the ages of 60 and 70 years. The decrease in IOP with age in the oldest age groups was still observed even after including participants receiving IOP-lowering medication, reducing the chance that the association is a result of bias due to participants with the highest IOP being excluded in older age due to commencement of therapy. If we assume that participants with higher IOP were more likely to undergo cataract surgery, it remains a possibility that the decline in IOP with age in people older than 70 years is due to exclusion of pseudophakic participants.

The reported association between IOP and sex is also inconsistent between studies; most studies (not included in the current meta-analysis) have reported higher IOP in women [15, 17, 18, 21, 22], though higher IOP in men [5, 16] or no association between IOP and sex have also been reported [19]. We found higher IOP in men, but only in adjusted analyses, and not in the subset with CCT available for further adjustment. This inconsistency raises the possibility of a chance finding. While higher IOP in men is in agreement with a higher risk of POAG in men [24], it is possible that a higher prevalence of angle-closure in women [25] also contributes to a sex-differential for IOP; iridocorneal drainage angle width may be an important determinant of IOP, even among healthy participants.

We found a significant decrease in IOP with greater height, even after adjustment for possible confounders. This is a relatively novel finding; while a negative crude association of height with IOP was reported in the Tanjong Pagar Study, this was not significant after adjustment for confounders [14]. Our finding is in agreement with the lower prevalence of POAG reported in taller participants of the Beijing Eye Study [26]. The mechanism underlying lower IOP in taller people is not clear, but may be related to the distance between the eye and the heart. We hypothesise that ciliary body perfusion and resultant aqueous production is lower the higher the eye is above the heart, and that this distance is larger in taller people. This is in agreement with the findings that IOP is lower in the sitting position compared with supine [27], and that IOP is lower in the higher eye of study participants in the lateral decubitus position [28].

The significant associations we found between IOP and BMI, SBP and spherical equivalent are consistent with the literature. The majority of published studies have reported higher IOP with higher BMI [15, 17–20, 22, 29, 30], higher SBP [14, 15, 17, 18, 20, 22, 31, 32], and more myopic refraction [23, 30] or longer axial length [20]. We have further examined the shapes of these relationships with IOP and found linear associations for BMI, SBP and spherical equivalent (Fig. 2). The linear relationship between BMI and IOP across the whole range of BMI is of particular interest. It has been suggested that the relationship between BMI and IOP is due to artefactual high IOP readings in people of high BMI due to an induced Valsalva manoeuvre at slit lamp examination [33]. However, our findings of higher IOP with BMI even at the lower end of the BMI range argue against the Valsalva hypothesis. For example, it would not be expected that a participant of normal BMI would have a greater degree of Valsalva manoeuvre induced at slit lamp examination than an underweight participant. Furthermore, the association between BMI and IOP was seen in studies using NCT, which may be less prone to inducing a Valsalva manoeuvre. The mechanism by which higher BMI increases IOP remains unclear, but may be related to metabolic syndrome in general [34]. A meta-analysis of epidemiological data suggests an increased risk of glaucoma in myopic people [35]. Higher IOP in myopic eyes may be the mechanism by which glaucoma risk is increased. What remains unclear is why IOP is higher in myopic eyes. A possible hypothesis is that abnormal elongation of the eye is associated with a degree of malformation of drainage angle microstructure.

We did not find striking variability of IOP levels between the European countries participating in this study, and did not find any variation in IOP with latitude. This may be in part due to relative genetic and cultural homogeneity among the predominantly Caucasian populations in this study, and in contrast to the significant difference seen in IOP of Japanese people compared with Europeans [36]. It is also likely that between study heterogeneity in IOP ascertainment limits meaningful comparisons of absolute IOP values, and reduces statistical power to identify small differences. One such difference in study methods is GAT versus NCT, and while we did repeat analyses stratified by tonometry method, the number of studies within each group was small and limited power for finding any differences. Furthermore, our studies did not represent a large range in latitude and were not entirely representative of Northern (e.g. lacking studies from Scandinavia) and Southern Europe. Future work combining studies in a larger global consortium may be better able to detect an association between IOP and latitude. Despite our negative findings, and the limitations of this approach, comparing IOP levels between countries remains an important method of potentially identifying new environmental associations with IOP.

The major strengths of our study are the large pooled sample size allowing identification of small effect associations, and the increased generalisability derived from demonstrating associations across multiple populations. Many epidemiological studies are limited by the possibility of chance findings or that findings are only relevant in the reported population. We have reported associations that were present when considering data from six different countries together, and could also examine the results from each study alone in relation to the pooled findings using the Forest plots. We can therefore be more certain that our results were not due to chance, and are likely applicable to many Caucasian populations within and outside Europe. There are also limitations to our study. Meta-analysis of summary data is a useful approach, but post hoc analysis is limited by the pre-specified analysis compared with pooling of raw data. However, the feasibility of sharing raw participant data between studies is limited by local study ethics arrangements. Another issue with meta-analysis is between study heterogeneity, which can limit the validity of statistically combining results. The degree of heterogeneity in the meta-analyses we conducted was variable, with I^2^ statistics ranging from 0 to 98 %. While random effects meta-analysis assumes a distribution of the true effect due to between study heterogeneity, it may not always be appropriate to statistically combine results from studies that used vastly different methods. For this reason, we also ran analyses for the major associations (Table 2; Fig. 1) stratified by tonometry method (GAT studies and NCT studies separately); this yielded very similar results (data not shown). While absolute IOP values may vary between GAT and NCT, the direction and strength of association of measured IOP with systemic factors did not appear to differ significantly. Another limitation is that Eastern European populations were not represented in our study sample. The cross-sectional nature of our data may limit causal inference for the associations detected.

In summary, novel findings from this large pan-European study included an inverted-U shaped association of IOP with age, and lower IOP in taller participants. We did not find significant variation in IOP across Europe. Our findings have implications for the design of future studies seeking novel aetiological factors for IOP, such as genetic association studies; depending on the study age-range, linear adjustment for age may not be appropriate, and pooling of data from studies of people of European descent may be appropriate given the lack of variation in IOP we have observed across Europe.

## Electronic supplementary material

Below is the link to the electronic supplementary material.

Supplementary material 1 (DOCX 45 kb)

Supplementary material 2 (DOCX 69 kb)

Supplementary material 3 (DOCX 7616 kb)

Supplementary material 4 (DOCX 136 kb)
