# Supplementary material for: Associations with intraocular pressure across Europe: The European Eye Epidemiology (E3) Consortium
Source: Eur J Epidemiol. 2016 Sep 9;31(11):1101–11. doi: 10.1007/s10654-016-0191-1 (PMC5206267; doi:10.1007/s10654-016-0191-1)

**Supplementary Figure 1:** Forest plots for standardized intraocular pressure (IOP), stratified by tonometry type and latitude. GAT – Goldmann applanation tonometry, NCT – non-contact tonometry.


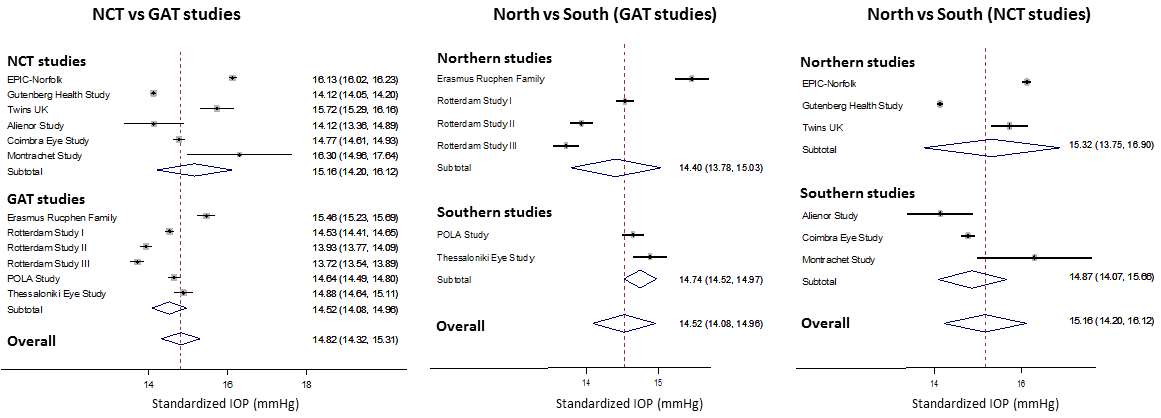

Supplement: Supplementary file 1 — Supplementary material 1 (DOCX 45 kb) [file 10654_2016_191_MOESM1_ESM.docx]
