# Supplementary material for: Associations with intraocular pressure across Europe: The European Eye Epidemiology (E3) Consortium
Source: Eur J Epidemiol. 2016 Sep 9;31(11):1101–11. doi: 10.1007/s10654-016-0191-1 (PMC5206267; doi:10.1007/s10654-016-0191-1)
Supplement: Supplementary file 2 — Supplementary material 2 (DOCX 69 kb) [file 10654_2016_191_MOESM2_ESM.docx]

**Supplementary Figure 2:** Meta-regressions for the association between latitude and standardized intraocular pressure (IOP), stratified by tonometry method. GAT – Goldmann applanation tonometry, NCT – non-contact tonometry.

**
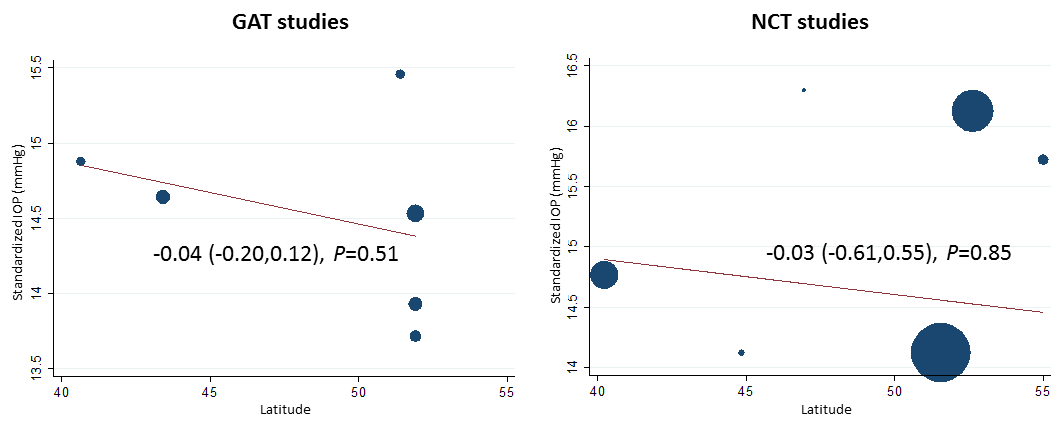
**
