# Supplementary material for: Associations with intraocular pressure across Europe: The European Eye Epidemiology (E3) Consortium
Source: Eur J Epidemiol. 2016 Sep 9;31(11):1101–11. doi: 10.1007/s10654-016-0191-1 (PMC5206267; doi:10.1007/s10654-016-0191-1)
Supplement: Supplementary file 3 — Supplementary material 3 (DOCX 7616 kb) [file 10654_2016_191_MOESM3_ESM.docx]

**Supplementary Section A – Study descriptions and IOP distributions**

**The Alienor study**

The Alienor (Antioxydants, Lipides Essentiels, Nutrition et maladies OculaiRes) Study is a population-based prospective study aiming at assessing the associations of age-related eye diseases (age-related maculopathy, glaucoma, cataract, dry eye syndrome) with nutritional factors (in particular antioxidants, macular pigment and fatty acids), determined from plasma measurements and estimation of dietary intakes. It also takes into account other major determinants of eye diseases, including gene polymorphisms, environmental factors and vascular factors. The methods of this study have been published elsewhere.([1](#_ENREF_1))

Subjects of the Alienor Study were recruited from an ongoing population-based study on the vascular risk factors for dementia, the Three-City (3C) Study.([2](#_ENREF_2)) The 3C Study included 9,294 subjects aged 65 years or more from three French Cities (Bordeaux, Dijon and Montpellier), among whom 2,104 were recruited in Bordeaux. They were initially recruited in 1999-2001 and followed-up about every two years since. The Alienor Study consists of eye examinations, which are proposed to all participants of the 3C cohort in Bordeaux since the third follow-up (2006-2008). Among the 1,450 participants re-examined between October 2006 and May 2008, 963 (66.4%) participated in the Alienor Study’s baseline eye examination. The design of this study has been approved by the Ethical Committee of Bordeaux (Comité de Protection des Personnes Sud-Ouest et Outre-Mer III) in May 2006.

Intraocular pressure (IOP) was measured with pneumotonometer (KT 800, Kowa, Japan). Central corneal thickness (CCT) was measured using Pachpen (Accutome Inc., Malvern Pa, USA). Refraction was measured using autorefractometer (Speedy K, Luneau, France) and refined subjectively when measuring best-corrected visual acuity. Cataract surgery was ascertained by the absence of the natural lens at slit lamp. Blood pressure was measured after the participant had been seated for at least 5 minutes. Systolic blood pressure was measured twice on the right arm using a digital electronic tensiometer (OMRON M4, France). The mean of two values was used for the analysis.

IOP distribution for Alienor Study:

**Coimbra Eye Study**

The Coimbra Eye Study is a cross-sectional, single-center, population-based study. The study was approved by the ethics committee. Between August 2009 and April 2011, subjects aged 55 years or older were recruited from the primary healthcare center of Mira. All participants underwent complete bilateral ophthalmologic examination. Best-corrected visual acuity (BCVA) was tested in each eye separately using the Early Treatment Diabetic Retinopathy Study (ETDRS) chart. If the BCVA of either eye was less than logMAR 0.2 refraction was performed with an autorefractor - NIDEK TONOREF II (autoref/kerato/tonometer), and the amended BCVA was recorded. Evaluation also included anterior segment biomicroscopy, tonometry with the same NIDEK TONOREF II (autoref/kerato/tonometer), and colour fundus photography, after pharmacological mydriasis. Two 35° non-simultaneous stereoscopic color fundus photographs were taken from fields 1M (centered on the optic disc), 2 (centered on the macula) and 3M (temporal to the macula), using a digital mydriatic Topocon fundus camera (TRC-50EX; Topcon Corporation, Tokyo, Japan). Fundus reflex photographs were similarly obtained to document media opacities.


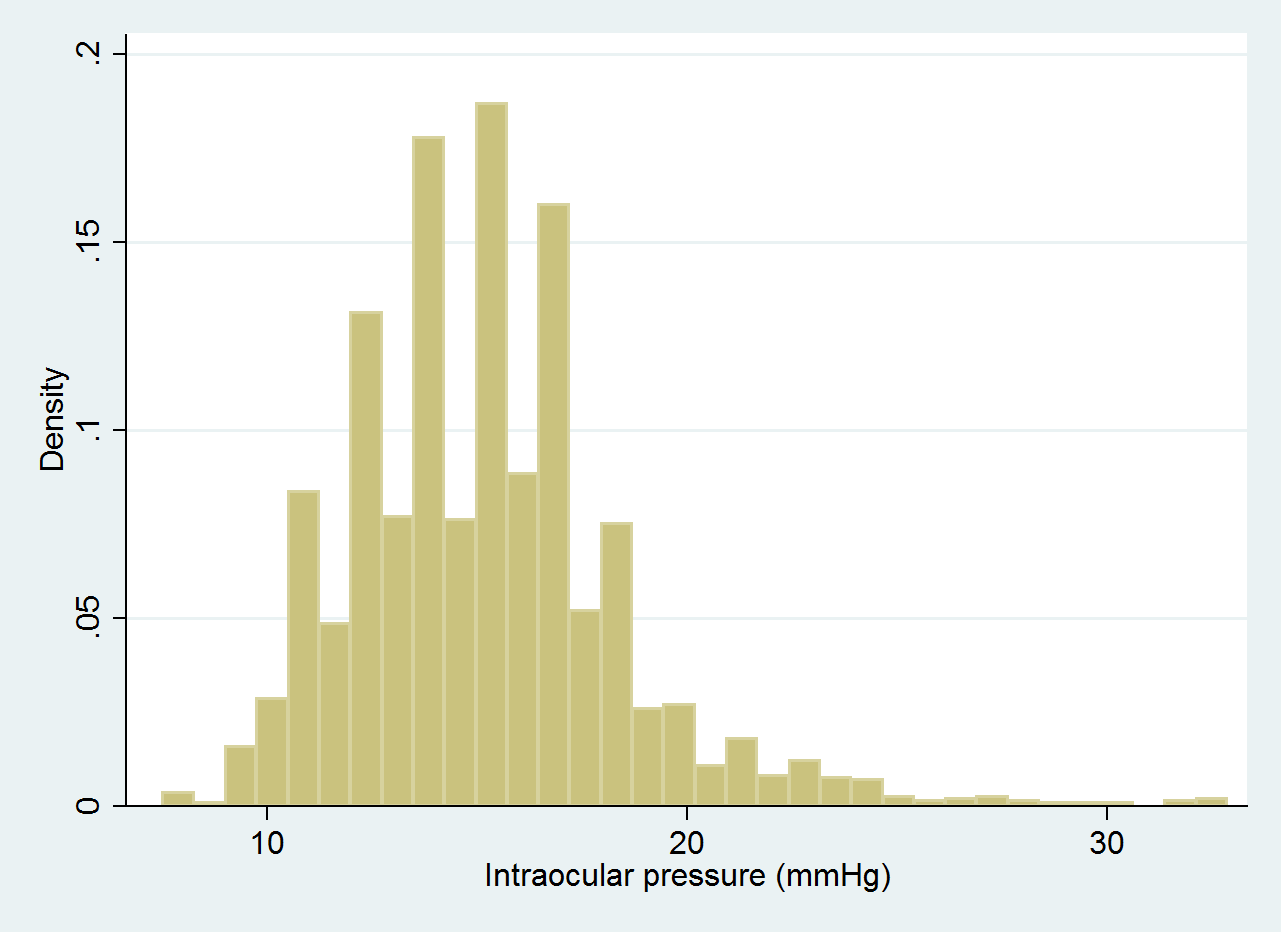


**EPIC-Norfolk Eye Study**

The European Prospective Investigation into Cancer (EPIC) study is a pan-European prospective cohort study designed to investigate the aetiology of major chronic diseases.([3](#_ENREF_3)) EPIC-Norfolk , one of the UK arms of EPIC, recruited and examined 25,639 participants aged 40-79 years between 1993 and 1997 for the baseline examination.([4](#_ENREF_4)) Recruitment was via general practices in the city of Norwich and the surrounding small towns and rural areas, and methods have been described in detail previously.([5](#_ENREF_5)) Since virtually all residents in the UK are registered with a general practitioner through the National Health Service, general practice lists serve as population registers. Ophthalmic assessment formed part of the third health examination and this has been termed the EPIC-Norfolk Eye Study.([6](#_ENREF_6)) In total, 8,623 participants were seen for the ophthalmic examination, between 2004 and 2011. The EPIC-Norfolk Eye Study was carried out following the principles of the Declaration of Helsinki and the Research Governance Framework for Health and Social Care. The study was approved by the Norfolk Local Research Ethics Committee (05/Q0101/191) and East Norfolk & Waveney NHS Research Governance Committee (2005EC07L).

IOP was measured using a non-contact instrument, the Ocular Response Analyser (ORA; Reichert, Corp., Buffalo, NY). Three readings were taken per eye and the best signal value of the Goldmann-correlated parameter used (based on the best quality pressure waveform as assessed by the ORA software). Height and weight were measured with participants wearing light clothing and no shoes. Height was measured to 0.1 cm using a stadiometer, and weight was measured to the nearest 0.1 kg using digital scales (Tanita UK Ltd., Middlesex, UK). Body mass index was calculated as weight/height^2^. Blood pressure was measured with the participant seated resting using an objective measurement device (Accutorr Plus; Datascope Patient Monitoring, Mindray UK, Ltd., Huntington, UK) on two separate occasions during the health examination and the mean of the two measurements considered. Refractive error was measured using a Humphrey Auto-Refractor 500 (Humphrey Instruments, San Leandro, California, USA). Central corneal thickness was measured using ultrasound pachymetry in a subset of participants meeting referral criteria for a hospital clinic examination (Pachmate DGH 55; DGH Technology, Exton, PA; mean of 10 readings per eye).

IOP distribution for EPIC-Norfolk Eye Study:


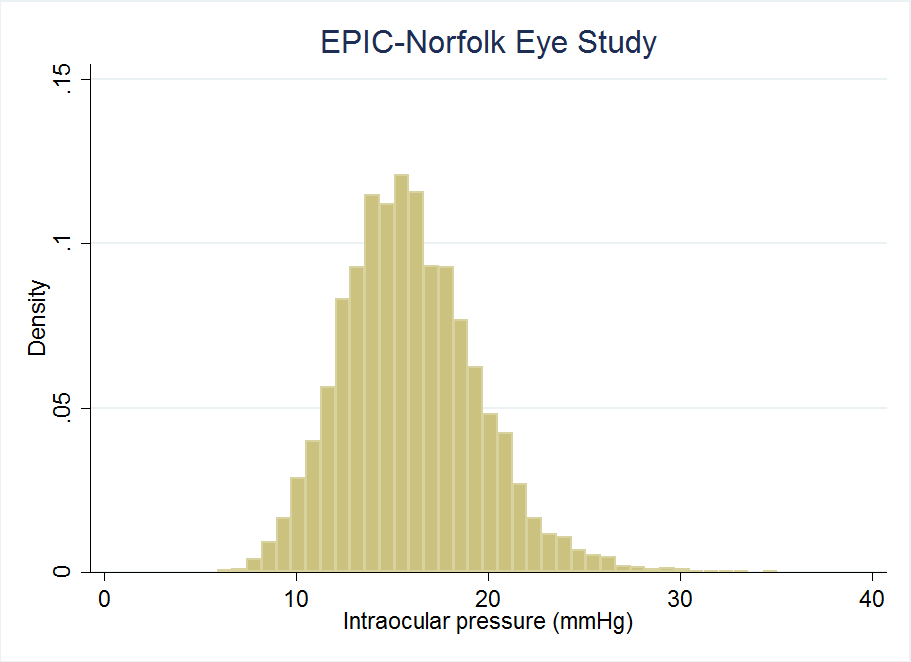


**Erasmus Rucphen Family (ERF) Study**

The ERF Study is a family-based cohort in a genetically isolated population in the southwest of the Netherlands with over 3,000 participants aged between 18 and 86 years. The rationale and study design of this study have been described elsewhere.([7](#_ENREF_7), [8](#_ENREF_8)) Cross-sectional examination took place between 2002 and 2005. IOP was measured with Goldmann applanation tonometry (Haag-Streit, Bern, Switzerland). IOP was measured twice per eye. If the two measurements in one eye differed, a third measurement was performed, and the median value was recorded. Refractive error was measured using a Topcon RM‐A2000 autorefractor (non-dilated). Height and weight were measured with the participant in light underclothing. Blood pressure was measured twice on the right arm in a sitting position after at least 5 min rest using an automated device (OMRON 711). The average of the 2 measures was used in the analyses. All measurements in these studies were conducted after the Medical Ethics Committee of the Erasmus University had approved the study protocols.

IOP distribution for ERF Study:

**Gutenberg Health Study**

The Gutenberg Health Study (GHS) is an ongoing, population-based, prospective, observational cohort study in the Rhine-Main Region in midwestern Germany with a total of 15,010 participants.([9](#_ENREF_9)) The study sample was recruited from subjects aged between 35 and 74 years at the time of the examination. Exclusion criteria were insufficient knowledge of the German language to understand explanations and instructions, and physical or psychic inability to participate in the examinations in the study center.

All participants underwent an ophthalmological investigation of 25 minutes’ duration taking place between 11:00 a.m. and 8:00 p.m. The IOP measurement was performed with a non-contact tonometer with automatic air-puff control (Nidek NT-2000™, Nidek Co., Japan).([10](#_ENREF_10)) The mean of three measurements within a range of 3 mmHg was obtained for each eye. Refractive error was measured non-dilated using a Humphrey® Automated Refractor/Keratometer (HARK) 599 (Carl Zeiss Meditec, Jena, Germany). Central corneal thickness was measured by optical pachymetry (Scheimpflug imaging with the Pachycam™, Oculus, Wetzlar, Germany). The measurement with the best quality (at least above 90%) per eye was selected for analysis.

The systolic blood pressure was determined as mean value of two standardized measurements (Omron HEM 705-CP II, OMRON, Mannheim, Germany) after 8 and 11 minutes of rest. Calibrated digital scales (Seca 862, Seca Germany) and a measuring stick (Seca 220, Seca, Germany) were used to take anthropometric measurements.

The study was approved by the Medical Ethics Committee of the state chamber of physicians of Rhineland-Palatinate and by the local and federal data safety commissioners.

IOP distribution for Gutenberg Health Study:


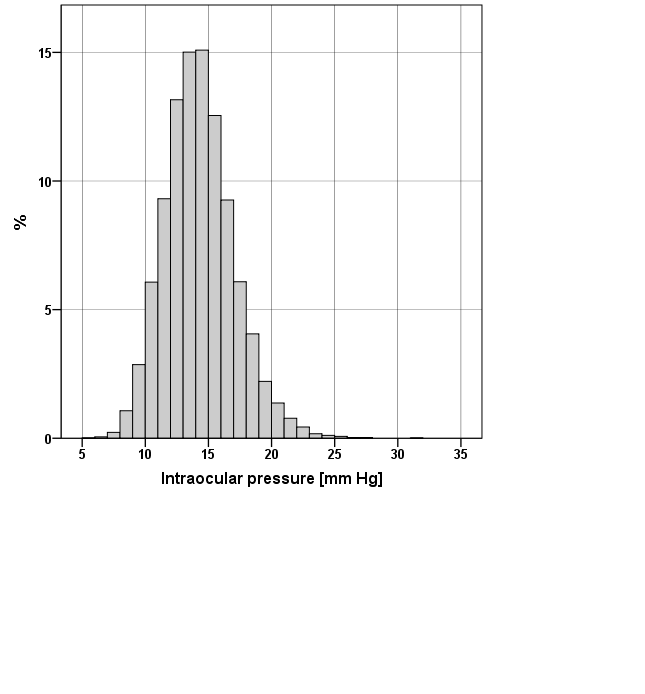


**The Montrachet 3C Study**

Subjects of the Montrachet (Maculopathy Optic Nerve nuTRition neurovAsCular and HEarT diseases) study were recruited from an ongoing population-based study, the Three-City (3C) study, on the vascular risk factors for dementia.([2](#_ENREF_2)) The 3C-Study was designed to examine the relationship between vascular diseases and dementia in a community housing 9,294 persons aged 65 years and over. The participants were selected from the electoral rolls and were only urban since they lived in 3 French cities, Bordeaux, Dijon and Montpellier. The 3C-Study began in 1999 and participants were evaluated every two years. A subgroup underwent ocular assessment in Bordeaux (Alienor study; see above)([1](#_ENREF_1)) and Dijon (Montrachet study).

In Dijon 4,934 subjects participated to the first run of the 3C-Study in 1999. They were followed every 2 years and at the fourth run undertaken in 2006/2007 they were still 3,137. Among them, 1,604 (51.1%) underwent an MRI at baseline and at the fourth year. We decided to include preferentially the participants having had an MRI and to complete the recruitment with participants without MRI. Therefore from October 22th, 2009 until March 31th, 2013, 913 volunteers with an MRI were recruited in the Montrachet study and 236 without and MRI. After approval by the regional ethics committee, the study was registered as 2009-A00448-49.

Intraocular pressure was measured by air tonometry (Tonoref II, Nidek, Aichi, Japan) and CCT was measured with an ultrasonic contact pachymeter (DGH 500, DGH Technology, Exton, PA, USA); the mean of 3 measurements was recorded for each eye. Refractive error was determined using an autorefractor without cycloplegia (Tonoref II, Nidek, Aichi, Japan). Height and weight were measured with participants wearing light clothes and no shoes. Systolic and diastolic blood pressures were recorded with a sphygmomanometer with participants resting seated for five minutes.

IOP distribution for Montrachet Study:


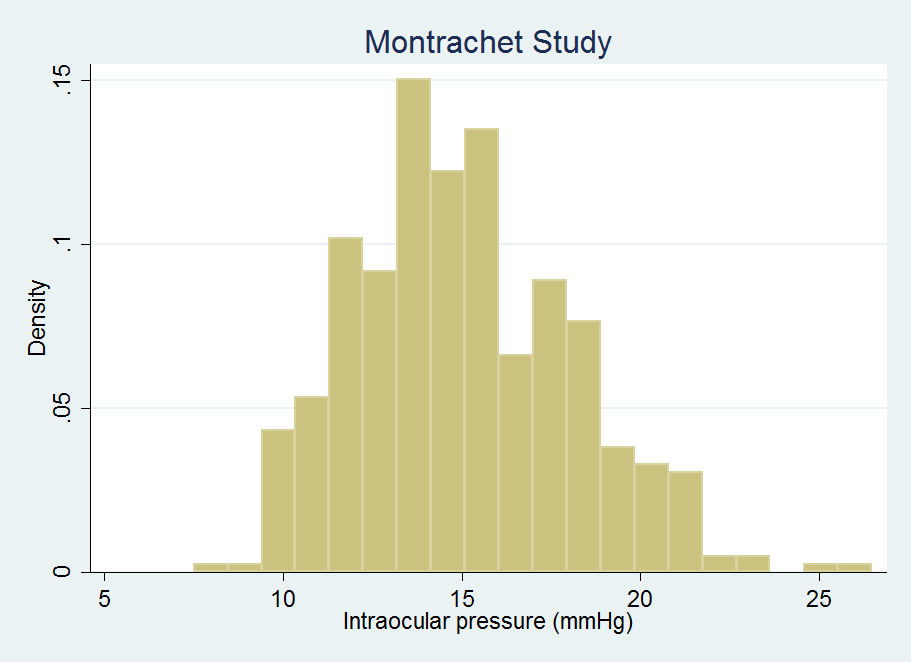


**The POLA Study**

The Pathologies Oculaires Liées à l’Age (POLA) Study is a population-based study aimed at identifying the risk factors of age-related eye diseases. The methods of this study have been published elsewhere.([11](#_ENREF_11)) For inclusion in the study, participants needed to be a resident of Sète (South of France) and aged 60 years and over. According to the 1990 population census, there were almost 12,000 eligible residents, of whom our objective was to recruit 3,000. The population was informed of the study through the local media. We also contacted 4,543 residents individually by mail and telephone, using the electoral roll. The baseline examinations took place in a mobile unit equipped with ophthalmologic devices. Between June 1995 and July 1997, 2,584 participants were recruited. The study was approved by the ethics committee of the University Hospital of Montpellier, France.

One IOP measurement was performed with Goldmann applanation tonometry in each eye. Refractive error was measure using a Topcon RM-A7000 autorefractor, and refined subjectively when assessing best-corrected visual acuity. Systolic and diastolic blood pressures were measured at the right arm after the participant had been seated for at least 5 minutes. Cataract surgery was ascertained by the absence of the natural lens at slit lamp.

IOP distribution for POLA Study:

**The Rotterdam Study I/II/III**

The Rotterdam Study is a population-based study established in Rotterdam, the Netherlands.([12](#_ENREF_12)) It consists of three cohorts. The original cohort, RS-I, started in 1990 and includes 7,983 subjects aged 55 years and older. The second cohort, RS-II, was added in 2000 and includes 3,011 subjects aged 55 years and older. The last cohort, RS-III, includes 3,932 subjects of 45 years of age and older and started in 2006. There is no overlap of participants between the studies. In all three cohorts, IOP was measured for both eyes with Goldmann applanation tonometry (Haag-Streit, Bern, Switzerland). The measurement was done twice. If the second measurement was different from the first measurement, a third measurement was performed and the median of all three values was taken. Refractive error was measured using a Topcon RM‐A2000 autorefractor (non-dilated). A subset of participants from RS-I underwent CCT measurements at baseline using ultrasound pachymetry (Allergan Humphrey 850, Carl Zeiss Meditec, Dublin, CA, USA). Another subset of participants from RS-I, RS-II and RS-III underwent CCT measurements at follow-up using a non-contact biometer (Lenstar LS900, Haag-Streit, Köniz, Switzerland). Height and weight were measured with indoor clothing and no shoes. Blood pressure was measured after the participant had been seated for at least 5 minutes. Systolic blood pressure was measured twice on the right arm using a random-zero sphygmomanometer with a 14x38 cm cuff. The mean of two values was used for the analysis. Other ophthalmic baseline and follow-up examinations, which are still ongoing, were described previously.([13](#_ENREF_13)) The Rotterdam Study has been approved by the institutional review board (Medical Ethics Committee) of the Erasmus Medical Center and by the review board of The Netherlands Ministry of Health, Welfare and Sports.

IOP distribution for Rotterdam Study I:

IOP distribution for Rotterdam Study II:

IOP distribution for Rotterdam Study III:

**Thessaloniki Eye Study**

The Thessaloniki Eye Study (TES) is a cross-sectional, population-based, epidemiologic study of chronic eye diseases in the Greek population of Thessaloniki. According to the National Statistical Service of Greece, Thessaloniki which is a major urban center in Northern Greece is considered representative of the general population in the country. The initial recruitment frame of the TES consisted of 5,000 people, 60 years of age or older, who were identified randomly in February 1999 from approximately 321,000 persons registered in the municipality registers of the city of Thessaloniki. Subject recruitment is described in detail elsewhere.([14](#_ENREF_14)) In summary, randomization was provided by the municipality statistical service. From the initial recruitment sample of the 5,000 names, 3,617 subjects were eligible and finally 2,554 participated in the study (participation rate 71%).([15](#_ENREF_15)) Study examination and data collection ended in March 2005. The study was approved by the Aristotle University Hospital Ethics Committee and the University of California Los Angeles Human Subject Protection Committee.

Visual acuity was measured with the Early Treatment of Diabetic Retinopathy Study (ETDRS) charts. If visual acuity was less than 20/30 with habitual correction, a full refraction was performed, and best-corrected visual acuity was measured. Intraocular pressure (IOP) was measured using a calibrated Goldmann applanation tonometer (Haag-Streit, Bern, Switzerland). The mean IOP of three readings in each eye was defined as the pressure for that eye. Blood pressure (BP) was considered as the average of two readings taken with an automated sphygmomanometer (model 705CP; OMRON Matsusaka Co Ltd, Matsusaka City, Japan) at least five minutes apart in the same arm, with the cuff approximately level with the heart. The readings were obtained before instillation of mydriatic drops and after the participant was seated for 10 minutes. Somatometric data were also measured as part of the TES protocol: Height and weight were measured with participants wearing light clothing and shoes. Height was measured using a stadiometer, and weight was measured using a 351 TERRAIL digital scales. Central corneal thickness was measured using ultrasound pachymetry in a subset of participants (A-scan, Quantel Medical, France; mean of 5 readings per eye).

IOP distribution for TES:


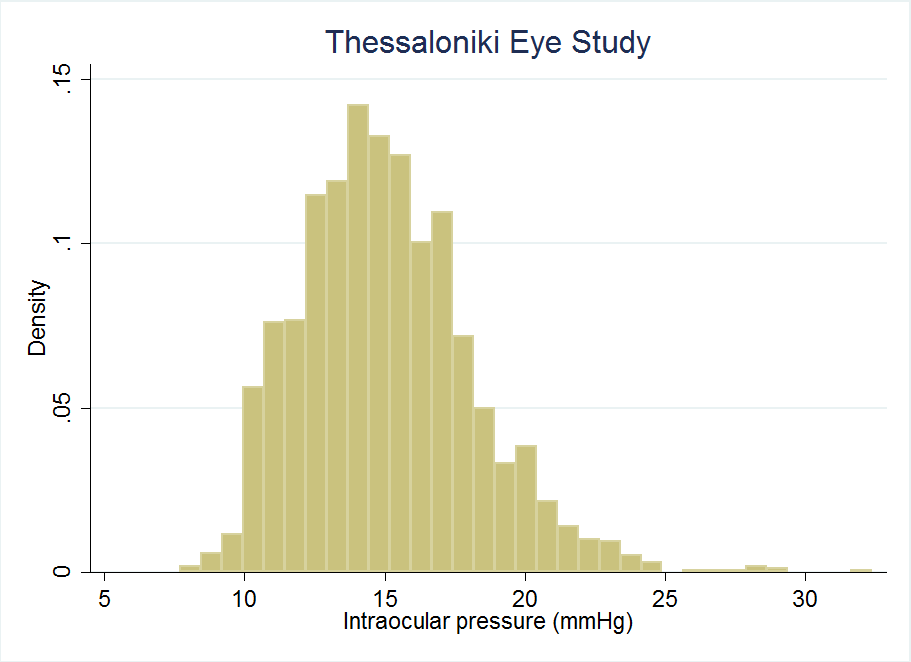


**TwinsUK**

The TwinsUK adult twin registry, based at St. Thomas' Hospital in London, compromises over 12,000 predominantly female Caucasian ancestry twins, from throughout the United Kingdom.([16](#_ENREF_16)) Twins largely volunteered unaware of the eye studies at the time of enrolment and gave fully informed consent under a protocol reviewed by the St. Thomas' Hospital Local Research Ethics Committee (EC04/015), which was performed in accordance with the Helsinki Declaration.

Various eye phenotypes have been collected on a subset of twins. IOP was measured between 2006 and 2010 with a non-contact air-puff tonometer, the Ocular Response Analyser (ORA, Reichert®, Buffalo, NY). The mean (Goldmann-equivalent) IOP was calculated from 4 readings (2 from each eye) for each participant; where quality indicators were poor or the two IOPs differed by more than 2mmHg, a third reading was taken. CCT was measured using an ultrasound pachymetry device provided with the ORA instrument. Refractive error was measured using non-cycloplegic autorefraction (ARM-10 autorefractor, Takagi Seiko, Japan). Blood pressure (measured three times with the automated Omron blood pressure machine, and the mean of second and third blood pressures used), height and weight were measured as part of other phenotype study visits. These examinations were not always performed on the same day as the eye examination, but in 87% of individuals measurements were recorded within a year of the eye examination. Body mass index was calculated as weight/height^2^.

IOP distribution for TwinsUK:


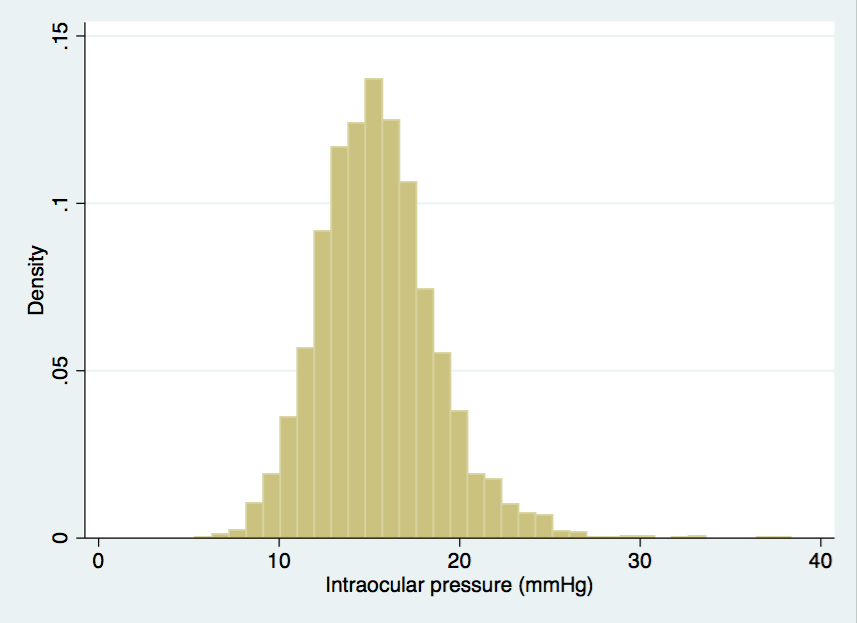


**References**

1. Delcourt C, Korobelnik JF, Barberger-Gateau P, Delyfer MN, Rougier MB, Le Goff M, et al. Nutrition and age-related eye diseases: the Alienor (Antioxydants, Lipides Essentiels, Nutrition et maladies OculaiRes) Study. The journal of nutrition, health & aging. 2010;14(10):854-61.

2. Group CS. Vascular factors and risk of dementia: design of the Three-City Study and baseline characteristics of the study population. Neuroepidemiology. 2003;22(6):316-25.

3. Riboli E, Kaaks R. The EPIC Project: rationale and study design. European Prospective Investigation into Cancer and Nutrition. International journal of epidemiology. 1997;26 Suppl 1:S6-14.

4. Day N, Oakes S, Luben R, Khaw KT, Bingham S, Welch A, et al. EPIC-Norfolk: study design and characteristics of the cohort. European Prospective Investigation of Cancer. British journal of cancer. 1999;80 Suppl 1:95-103.

5. Hayat SA, Luben R, Keevil VL, Moore S, Dalzell N, Bhaniani A, et al. Cohort profile: A prospective cohort study of objective physical and cognitive capability and visual health in an ageing population of men and women in Norfolk (EPIC-Norfolk 3). International journal of epidemiology. 2014;43(4):1063-72.

6. Khawaja AP, Chan MP, Hayat S, Broadway DC, Luben R, Garway-Heath DF, et al. The EPIC-Norfolk Eye Study: rationale, methods and a cross-sectional analysis of visual impairment in a population-based cohort. BMJ open. 2013;3(3).

7. Aulchenko YS, Heutink P, Mackay I, Bertoli-Avella AM, Pullen J, Vaessen N, et al. Linkage disequilibrium in young genetically isolated Dutch population. European journal of human genetics : EJHG. 2004;12(7):527-34.

8. Pardo LM, MacKay I, Oostra B, van Duijn CM, Aulchenko YS. The effect of genetic drift in a young genetically isolated population. Annals of human genetics. 2005;69(Pt 3):288-95.

9. Wild PS, Zeller T, Beutel M, Blettner M, Dugi KA, Lackner KJ, et al. [The Gutenberg Health Study]. Bundesgesundheitsblatt, Gesundheitsforschung, Gesundheitsschutz. 2012;55(6-7):824-9.

10. Hoehn R, Mirshahi A, Hoffmann EM, Kottler UB, Wild PS, Laubert-Reh D, et al. Distribution of intraocular pressure and its association with ocular features and cardiovascular risk factors: the Gutenberg Health Study. Ophthalmology. 2013;120(5):961-8.

11. Delcourt C, Diaz JL, Ponton-Sanchez A, Papoz L. Smoking and age-related macular degeneration. The POLA Study. Pathologies Oculaires Liees a l'Age. Archives of ophthalmology. 1998;116(8):1031-5.

12. Hofman A, Darwish Murad S, van Duijn CM, Franco OH, Goedegebure A, Ikram MA, et al. The Rotterdam Study: 2014 objectives and design update. European journal of epidemiology. 2013;28(11):889-926.

13. Wolfs RC, Borger PH, Ramrattan RS, Klaver CC, Hulsman CA, Hofman A, et al. Changing views on open-angle glaucoma: definitions and prevalences--The Rotterdam Study. Investigative ophthalmology & visual science. 2000;41(11):3309-21.

14. Topouzis F, Coleman AL, Harris A, Jonescu-Cuypers C, Yu F, Mavroudis L, et al. Association of blood pressure status with the optic disk structure in non-glaucoma subjects: the Thessaloniki eye study. American journal of ophthalmology. 2006;142(1):60-7.

15. Topouzis F, Wilson MR, Harris A, Anastasopoulos E, Yu F, Mavroudis L, et al. Prevalence of open-angle glaucoma in Greece: the Thessaloniki Eye Study. American journal of ophthalmology. 2007;144(4):511-9.

16. Moayyeri A, Hammond CJ, Hart DJ, Spector TD. The UK Adult Twin Registry (TwinsUK Resource). Twin research and human genetics : the official journal of the International Society for Twin Studies. 2013;16(1):144-9.

**The European Eye Epidemiology (E^3^) Consortium:**

| **First name** | **Last name** | **Institution** | **City** | **Country** |
| --- | --- | --- | --- | --- |
| Niyazi | Acar | Inra-University of Burgundy | Dijon | France |
| Eleftherios | Anastosopoulos | University of Thessaloniki | Thessaloniki | Greece |
| Augusto | Azuara-Blanco | Queen's University | Belfast | UK |
| Arthur | Bergen | Netherlands Institute for Neurosciences-KNAW | Amsterdam | Netherlands |
| Geir | Bertelsen | University of Tromso | Tromso | Norway |
| Christine | Binquet | University Hospital of Dijon | Dijon | France |
| Alan | Bird | Moorfield's Eye Hospital | London | UK |
| Lionel | Brétillon | Inra-University of Burgundy | Dijon | France |
| Alain | Bron | University Hospital of Dijon | Dijon | France |
| Gabrielle | Buitendijk | Erasmus Medical Center | Rotterdam | Netherlands |
| Maria Luz | Cachulo | AIBILI/CHUC | Coimbra | Portugal |
| Usha | Chakravarthy | Queen's University | Belfast | UK |
| Michelle | Chan | UCL Institute of Ophthalmology | London | UK |
| Petrus | Chang | University of Bonn | Bonn | Germany |
| Annemarie | Colijn | Erasmus Medical Center | Rotterdam | Netherlands |
| Audrey | Cougnard-Grégoire | University of Bordeaux Segalen | Bordeaux | France |
| Catherine | Creuzot-Garcher | University Hospital of Dijon | Dijon | France |
| Philippa | Cumberland | UCL Institute of Child Health | London | UK |
| José | Cunha-Vaz | AIBILI/CHUC | Coimbra | Portugal |
| Vincent | Daien | Inserm U1061 | Montpellier | France |
| Gabor | Deak | Medical University of Vienna | Vienna | Austria |
| Cécile | Delcourt | University of Bordeaux Segalen | Bordeaux | France |
| Marie-Noëlle | Delyfer | University of Bordeaux Segalen | Bordeaux | France |
| Anneke | den Hollander | Radboud University | Nijmegen | Netherlands |
| Martha | Dietzel | University of Muenster | Muenster | Germany |
| Maja Gran | Erke | University of Tromso | Tromso | Norway |
| Sascha | Fauser | University Eye Hospital | Cologne | Germany |
| Robert | Finger | University of Bonn | Bonn | Germany |
| Astrid | Fletcher | London School of Hygiene and Tropical Medicine | London | UK |
| Paul | Foster | UCL Institute of Ophthalmology | London | UK |
| Panayiota | Founti | University of Thessaloniki | Thessaloniki | Greece |
| Arno | Göbel | University of Bonn | Bonn | Germany |
| Theo | Gorgels | Netherlands Institute for Neurosciences-KNAW | Amsterdam | Netherlands |
| Jakob | Grauslund | University of Southern Denmark | Odense | Denmark |
| Franz | Grus | University Medical Center Mainz | Mainz | Germany |
| Christopher | Hammond | King's College | London | UK |
| Catherine | Helmer | University of Bordeaux Segalen | Bordeaux | France |
| Hans-Werner | Hense | University of Muenster | Muenster | Germany |
| Manuel | Hermann | University Eye Hospital | Cologne | Germany |
| René | Hoehn | University Medical Center | Mainz | Germany |
| Ruth | Hogg | Queen's University | Belfast | UK |
| Frank | Holz | University of Bonn | Bonn | Germany |
| Carel | Hoyng | Radboud University | Nijmegen | Netherlands |
| Nomdo | Jansonius | Erasmus Medical Center | Rotterdam | Netherlands |
| Sarah | Janssen | Netherlands Institute for Neurosciences-KNAW | Amsterdam | Netherlands |
| Anthony | Khawaja | UCL Institute of Ophthalmology | London | UK |
| Caroline | Klaver | Erasmus Medical Center | Rotterdam | Netherlands |
| Jean-François | Korobelnik | University of Bordeaux Segalen | Bordeaux | France |
| Julia | Lamparter | University Medical Center Mainz | Mainz | Germany |
| Mélanie | Le Goff | University of Bordeaux Segalen | Bordeaux | France |
| Sergio | Leal | AIBILI/CHUC | Coimbra | Portugal |
| Yara | Lechanteur | Radboud University | Nijmegen | Netherlands |
| Terho | Lehtimäki | Pirkanmaa Hospital District | Tampere | Finland |
| Andrew | Lotery | University of Southampton | Southampton | UK |
| Irene | Leung | Moorfield's Eye Hospital | London | UK |
| Matthias | Mauschitz | University of Bonn | Bonn | Germany |
| Bénédicte | Merle | University of Bordeaux Segalen | Bordeaux | France |
| Verena | Meyer zu Westrup | University of Muenster | Muenster | Germany |
| Edoardo | Midena | University of Padova | Padova | Italy |
| Stefania | Miotto | University of Padova | Padova | Italy |
| Alireza | Mirshahi | University Medical Center | Mainz | Germany |
| Sadek | Mohan-Saïd | Institut de la Vision | Paris | France |
| Alyson | Muldrew | Queen's University | Belfast | UK |
| Michael | Mueller | Pirkanmaa Hospital District | Tampere | Finland |
| Sandrina | Nunes | AIBILI/CHUC | Coimbra | Portugal |
| Konrad | Oexle | Institue of Human Genetics | Munich | Germany |
| Tunde | Peto | Moorfield's Eye Hospital | London | UK |
| Stefano | Piermarocchi | University of Padova | Padova | Italy |
| Elena | Prokofyeva | Inserm U1018 | Paris | France |
| Jugnoo | Rahi | UCL Institute of Ophthalmology | London | UK |
| Olli | Raitakari | Pirkanmaa Hospital District | Tampere | Finland |
| Luisa | Ribeiro | AIBILI/CHUC | Coimbra | Portugal |
| Marie-Bénédicte | Rougier | University of Bordeaux Segalen | Bordeaux | France |
| José | Sahel | Institut de la Vision | Paris | France |
| Aggeliki | Salonikiou | University of Thessaloniki | Thessaloniki | Greece |
| Clarisa | Sanchez | Radboud University | Nijmegen | Netherlands |
| Steffen | Schmitz-Valckenberg | University of Bonn | Bonn | Germany |
| Cédric | Schweitzer | University of Bordeaux Segalen | Bordeaux | France |
| Tatiana | Segato | University of Padova | Padova | Italy |
| Jasmin | Shehata | Medical University of Vienna | Vienna | Austria |
| Rufino | Silva | AIBILI/CHUC | Coimbra | Portugal |
| Giuliana | Silvestri | Queen's University | Belfast | UK |
| Christian | Simader | Medical University of Vienna | Vienna | Austria |
| Eric | Souied | University Hospital of Créteil | Créteil | France |
| Henriet | Springelkamp | Erasmus Medical Center | Rotterdam | Netherlands |
| Robyn | Tapp | Pirkanmaa Hospital District | Tampere | Finland |
| Fotis | Topouzis | University of Thessaloniki | Thessaloniki | Greece |
| Virginie | Verhoeven | Erasmus Medical Center | Rotterdam | Netherlands |
| Therese | Von Hanno | University of Tromso | Tromso | Norway |
| Stela | Vujosevic | University of Padova | Padova | Italy |
| Katie | Williams | King's College London | London | UK |
| Christian | Wolfram | University Medical Center | Mainz | Germany |
| Jennifer | Yip | UCL Institute of Ophthalmology | London | UK |
| Jennyfer | Zerbib | University Hospital of Créteil | Créteil | France |
| Isabella | Zwiener | University Medical Center | Mainz | Germany |
