# Supplementary material for: Associations with intraocular pressure across Europe: The European Eye Epidemiology (E3) Consortium
Source: Eur J Epidemiol. 2016 Sep 9;31(11):1101–11. doi: 10.1007/s10654-016-0191-1 (PMC5206267; doi:10.1007/s10654-016-0191-1)

**Supplementary section B – Influence Analysis**

Given the between study heterogeneity, we conducted an influence analysis that examined the contribution of each study to the heterogeneity by sequentially omitting one study and reanalysing the pooled estimate for the remaining studies. We have carried this out for the multivariable regressions containing age, sex, body mass index, height, systolic blood pressure and spherical equivalent in the same model (Model 1, Table 2). The pooled effect estimates with 95% confidence intervals are presented beside the name of the study that was omitted (i.e. the presented result is for a meta-analysis of all studies *except* the study named). The x axis in each graph represents change in intraocular pressure (mmHg) and the vertical blue lines represent the pooled effect estimate and 95% confidence intervals for all studies with no omission. An influence analysis was carried out for each variable in the multivariable regression model.

**Age (per decade older)**


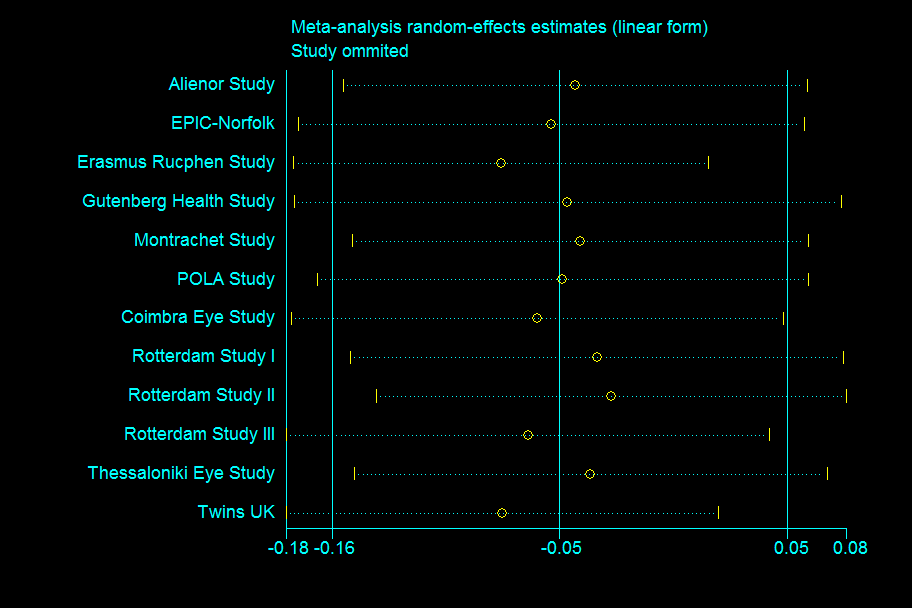


**Female sex**


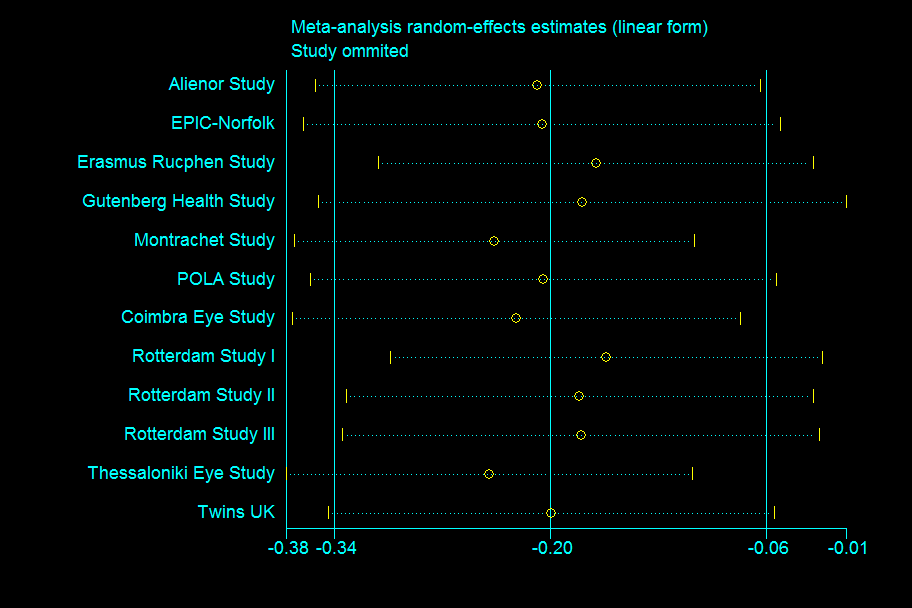


**Body mass index (per 5 Kg/m^2^)**


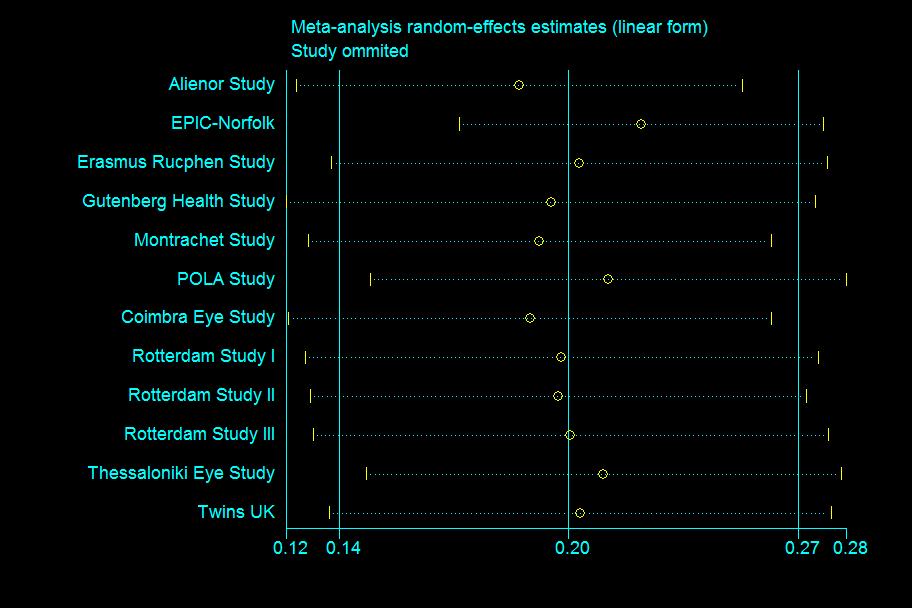


**Height (per 10 cm)**


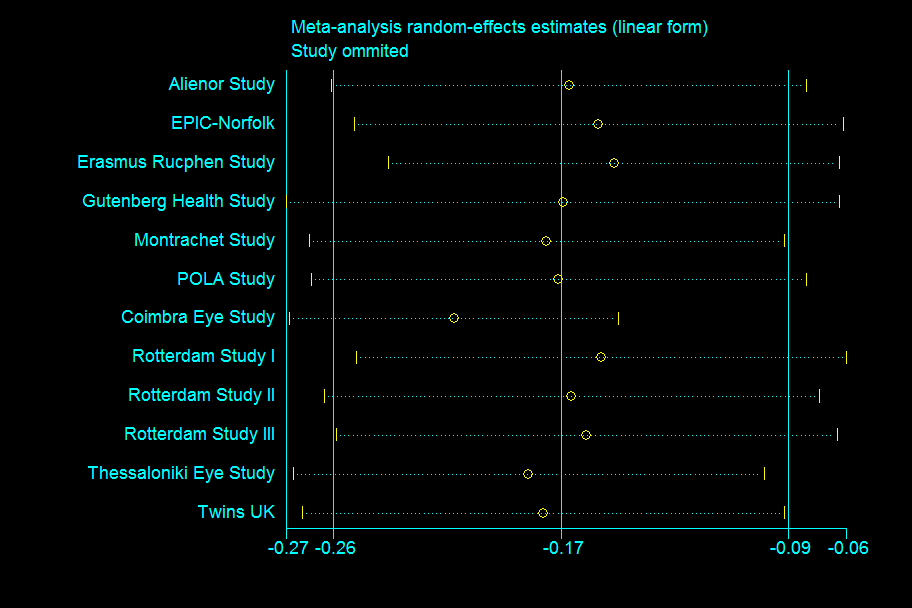


**Systolic blood pressure (per 10 mmHg)**


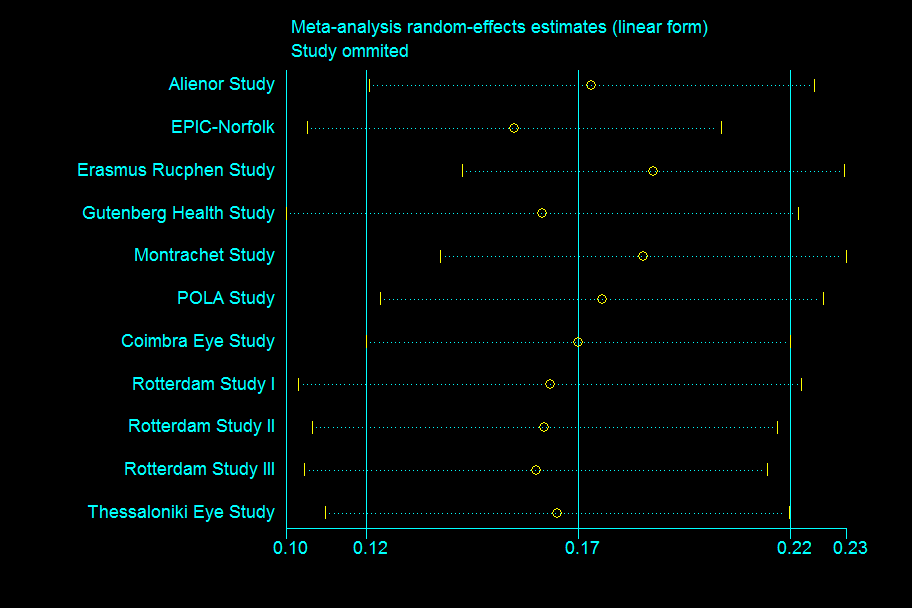


**Spherical equivalent (per dioptre)**


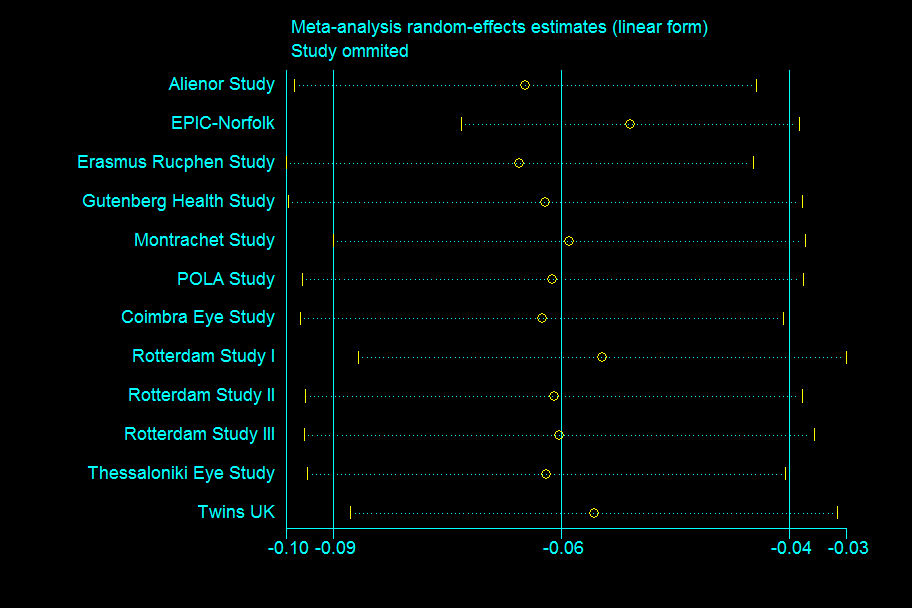

Supplement: Supplementary file 4 — Supplementary material 4 (DOCX 136 kb) [file 10654_2016_191_MOESM4_ESM.docx]
